# Validity and Reliability of the Psychodynamic Organizational Diagnostic Instrument SyMOA: Protocol for a Mixed Methods Study

**DOI:** 10.2196/77554

**Published:** 2026-03-05

**Authors:** Anne-Maria Müller, Sophia Sachs, Yannik Rieder, Claas Lahmann

**Affiliations:** 1 Department of Psychosomatic Medicine and Psychotherapy, Faculty of Medicine, University Medical Center Freiburg, Hauptstrasse 8, Freiburg, 79104, Germany, 49 761270 ext 68812

**Keywords:** organizational assessment, systems psychodynamics, psychodynamic theory, organizational health, validation

## Abstract

**Background:**

A comprehensive understanding of organizations is fundamental for implementing successful change measures. However, to date, there is no empirically testable, operationalized systems-psychodynamic organizational diagnostic method that can capture the deeper, more complex dynamics that are crucial for sustainable transformation. To address this gap, we developed the Systematic Multidimensional Organizational Assessment (SyMOA), a qualitative instrument based on an evidence-based clinical diagnostic framework, the Operationalized Psychodynamic Diagnostics III. SyMOA integrates clinical, organizational, and systemic psychodynamic theory and analyzes an organization’s challenges based on invisible and unconscious aspects, that is, those lurking beneath the surface. It hypothesizes 3 organizational dimensions: (1) current challenges based on the sociotechnical integration and organizational internal functioning level, (2) internal relationship dynamics, and (3) unconscious organizational conflicts. The SyMOA dimensions are operationalized into a semistructured interview guide and coding protocols for the analysis of the content. By capturing the underlying dynamics, SyMOA aims to provide a deeper understanding of an organization’s challenges and establish a solid foundation for targeted interventions.

**Objective:**

This study aims to evaluate the validity and intercoder reliability of the SyMOA instrument.

**Methods:**

For this purpose, semistructured interviews will be conducted with employees of at least 3 different companies in Germany. The evaluation will be carried out by calculating Krippendorff α to determine intercoder reliability. In addition, construct validity, content validity, and external validity will also be analyzed.

**Results:**

Recruitment and training commenced in May 2025. Data collection is planned for the second half of 2025, with analysis to follow thereafter. As this is a study protocol, no results are available yet. At the time of submission, 46 participants have been recruited.

**Conclusions:**

This study will give methodological insights into the validity, reliability, feasibility, and acceptability of the SyMOA instrument. The findings are expected to help further instrument refinement and inform the application of systems-psychodynamic approaches in organizational diagnostics.

## Introduction

### Background

Organizations are complex social systems that are shaped by the interaction of their members. These individuals bring their own history, personality, and emotions, which significantly influence the dynamics within an organization [1]. At the same time, organizations are in a constant state of change in order to adapt to external changes and secure their long-term existence [2]. Nevertheless, planned change processes are often slow, ineffective, and meet with resistance [3,4]. In addition, organizational employees experience change processes as a significant source of stress [4]. Resistance toward change and adaptation processes is common and signals the activation of—at least initially mainly unconscious—anxiety [5].

According to many researchers, the basis for meaningful and sustainable change management is a thorough and focused organizational diagnosis [3,4,6,7]. It is evident, though, that organizational structures and processes go far beyond the visible level, and models of organizational behavior based on behaviorist concepts have not been sufficient to look beneath the surface and identify the deep structure of underlying problems [8]. Systems psychodynamic research has contributed to understanding these hidden layers by addressing the interplay between collective structures, norms and practices, as well as individual cognitions, motivations, and emotions [1]. Systems psychodynamic theory combines the theory of open systems, which describes organizations as self-regulating systems that interact with their environment [1], with psychodynamic theory that understands organizational conflicts as unavoidable and structure-shaping elements. Psychodynamic theory postulates that the organization becomes a symbolic replica of the family in which members try to fulfill basic psychological needs. In this sense, organizational culture can be understood as an institutionalized defense mechanism that provides members of the organization relief from the anxiety caused by unavoidable change [5]. These social defense mechanisms are embedded in the organizational structure and thus influence the thinking, feeling, and behavior of its members [9].

### Literature Gap and Problem Statement

Theoretical and practice-oriented systems-psychodynamic approaches to organizational analysis are existent and well established [1,8,10]. Nevertheless, to date, there is no empirically testable, standardized, and operationalized diagnostic instrument capable of systematically assessing psychodynamic content within organizations while providing a structured procedure and a structured coding and interpretation protocol that translates systems-psychodynamic organizational theory into a transparent and reproducible diagnostic instrument.

The lack of an empirically testable and operationalized systems-psychodynamic diagnostic instrument is a problem for research and practice alike. Without this tool, unconscious conflict patterns, relational dynamics, organizational defenses, and the inner-organizational level of functioning cannot be analyzed in a structured, transparent, or reproducible manner. On the side of organizational practice, this limitation restricts organizations’ ability to understand the deeper causes of resistance to change and failed change initiatives. On the side of research, the lack of an operationalized instrument hinders the systematic empirical testing and comparison of findings in systems-psychodynamic organizational research.

### Prior Work

#### Overview

To close this gap, the authors of this article developed the Systematic Multidimensional Organizational Assessment (SyMOA, publication in process). SyMOA is a qualitative, systems-psychodynamic diagnostic instrument that aims to assess organizations based on the 3 dimensions of (1) current challenges analyzed on the basis of sociotechnical integration and internal level of functioning, (2) underlying relationship dynamics, and (3) unconscious organizational conflicts. SyMOA was developed by the authors using a theory-to-research approach based on clinical, psychodynamic, and organizational theories and was refined through an iterative process of theory development, empirical application in small- and medium-sized enterprises (SMEs), and theoretical refinement. The model from clinical practice that served as the basis for SyMOA’s in-depth organizational diagnostics is the Operationalized Psychodynamic Diagnostics III [11]. It enables a comprehensive analysis of psychodynamic processes and is successfully used for therapy planning [11,12]. Adapted and applied to organizations, such diagnostics can help to specifically support a company in understanding the causes of resistance and enable its ability to learn and adapt [13]. There is precedence for transferring psychological instruments, developed with the individual in mind, into the organizational context. Psychological concepts like emotional intelligence, motivation theories, and psychodynamic coaching have already been successfully adapted to organizations. Furthermore, both individuals and organizations exhibit complex psychological dynamics such as defense mechanisms, conflicts, and relational patterns [8]. SyMOA in its current version is a comprehensive and in-depth qualitative instrument that aims to capture systemic and psychodynamic aspects within an organization that no existing instrument has been able to map so far. It provides a semistructured interview procedure and a structured, operationalized rating system designed to enable psychodynamic assessment. This developmental work has been done separately and prior to the present study.

#### SyMOA Dimension I: Current Challenges Based in the Sociotechnical Integration and the Organization’s Internal Level of Functioning

The first dimension describes the organization’s understanding of current and past challenges, including its resources and barriers to change. Additionally, this dimension delves deeper beneath the surface to examine the organization’s internal level of functioning. This includes how the organization, as a system, perceives itself and its environment, how it regulates itself both internally and externally in everyday situations as well as in critical moments, how internal and external communication functions, and how well it establishes bonds—both internally among employees and externally with stakeholders such as customers, the market, and supply chains. Furthermore, this dimension captures the defense mechanisms typically used by the organization.

#### SyMOA Dimension II: Internal Relationship Dynamics of the Organization

The second dimension assesses the relationship dynamics between different groups within the organization. This includes relationship patterns in typical situations between individual employees, within teams, between teams, between management and stakeholders, and between management and employees. Relationship dynamics are increasingly seen as the key to overcoming nonroutine challenges. Assessment of the relational dynamics in the organization focuses on observable relational behavior and conscious emotional experience in specific situations. Of high importance for the assessment is the perspective of experience and how the specific constellations of perspectives influence (dys)functional patterns of relationships. It can shed light on recurring interaction patterns in organizations, where party X experiences itself and others (party Y) in a given situation, and how the other party (Y) experiences party X, as well as how party Y experiences itself in interaction with party X.

#### SyMOA Dimension III: Unconscious Organizational Conflicts

Based on psychodynamic theories of motivation, emotions, and unconscious conflicts, this dimension conceptualizes the internal conflicts within organizations. Typical conflicts include dependence versus autonomy, control versus subordination, intrinsic versus extrinsic organization-based self-worth conflicts, provision versus self-sufficiency, organizational exploitation versus prosocial engagement, market dominance, and identity conflicts. Conflicts are initially external, rooted in the early history of the company, but over time they become internalized and thus develop into unconscious internal conflicts. Conflict represents the clash of opposing positions, the collision of motives, desires, needs, values, and ideas. Conflicts between competing needs are a normal and everyday occurrence, such as the need for self-determination in the workplace on one hand and the desire for leadership and guidance on the other. If the underlying conflict is adequately resolved, individuals within the organization can shift flexibly between these needs depending on the situation. However, stress or dysfunction arises when the organization fails to develop an adequate strategy to resolve the fundamental conflict and persistently tries to rigidly implement one pole of these needs. These conflicts are repeatedly triggered by situational factors throughout the organization’s development.

### Research Objectives

SyMOA has been developed from a theory-driven perspective and has been refined through preliminary applications in 2 SMEs. To date, SyMOA has not been applied outside of its developmental testing. Furthermore, the psychometric properties and methodological robustness have not yet been empirically evaluated. There is still a lack of evidence on the instrument’s reliability, validity, feasibility, and acceptability. For SyMOA to become established as a scientifically sound diagnostic instrument and to be used confidently in organizational practice, this gap needs to be closed. Specifically, the study examines (1) the intercoder reliability of the SyMOA rating system; (2) the construct, content, and external validity of the instrument; and (3) the feasibility and acceptability of its interview and coding procedures in organizational practice. The present study therefore constitutes the first systematic evaluation of SyMOA’s reliability and validity.

## Methods

### Ethical Considerations

The study was approved by Ethikkommission Universitätsklinikum Freiburg on March 3, 2025, under the trial ID 24-1561-S2. The study is being conducted in accordance with the local legislation and institutional requirements. The participants will provide their written informed consent to participate in this study. When presenting data from the interviews, particular care will be taken to ensure that published statements—especially those concerning conflict situations—do not negatively impact individuals or the company. Consent for publication is included in the informed consent process. The trial was registered with the Freiburger Clinical Trials Register (Freiburger Register Klinischer Studien; trial ID FRKS005508) on December 17, 2024.

Participants’ privacy and confidentiality will be fully respected. No identifying personal information will be collected. Interviews will focus exclusively on participants’ perspectives on organizational dynamics and working relationships. While participants may refer to personal experiences, all data will be analyzed and reported in an anonymized form.

Audio recordings will be stored without personal identifiers using encrypted storage systems and will be accessible only to the research team. The recordings will not be shared with third parties and will not be publicly available. No images or visual materials of participants will be collected or included. The study does not involve patients or clinical data.

No financial or nonfinancial compensation will be provided to participants for taking part in the study.

### Objectives, Design, and Setting

The aim of the study is to determine intercoder reliability (Krippendorff α) and validity (construct, content, and external validity) of the newly designed SyMOA instrument. The study follows an exploratory qualitative multicase research design aiming to recruit at least 3 SMEs.

### Study Population

The study focuses on SMEs in the industrial sector in Germany. Organizations with fewer than 30 employees are excluded. Participants within the selected organization must meet the following criteria: (1) aged above 18 years, (2) with the company for more than 1 month, and (3) proficient in German. Employees who do not meet these criteria will be excluded. Factors such as age, gender, and social status will not be a priority in the selection, as all employees in the respective critical department of the company will be approached. Sociodemographic data will be documented.

### Study Procedure

**Figure 1.**
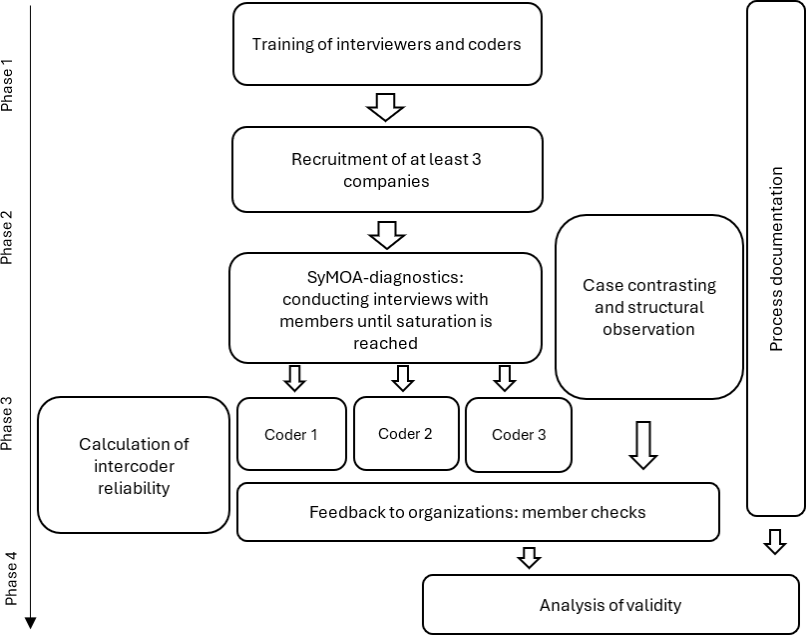
Overview of the study procedure. SyMOA: Systematic Multidimensional Organizational Assessment.

The study flow is illustrated in Figure 1.

The first phase will be dedicated to training of the researchers and coders in the interview guide and rating criteria. The second phase will focus on recruitment of interested organizations and obtaining informed consent from the organization’s management and participating employees. This will be followed by data collection, including semistructured interviews supplemented by structured on-site observations documented in a research diary (logbook with templates). If necessary, follow-up data collection may be arranged with the organizations. Three researchers will independently code the interviews using the audio recordings and the transcripts using MAXQDA24 (VERBI Software) [14]. The final phase will be dedicated to data analysis and reporting, culminating in oral and written feedback to the participating companies, including recommendations for potential interventions.

### Training of Raters

All interviewers and raters are qualified psychologists. The training was carried out by 1 of the writers of the SyMOA framework. The training contains 2 sessions of 2.5 hours each and interim exercises. The first session focuses on imparting the theoretical buildup of the interview guide, the questioning process, operationalization of the constructs, and their rating. The basis is a logical understanding of the content, followed by an exploration of psychological meaning through the examination of contradictions and experiential context. A scenic understanding to assess interactional patterns and organizational culture, culminating in the interpretation of unconscious elements will be trained. While this interpretative process remains open to emergent themes, it is guided by the predefined dimensions of SyMOA and incorporates interpretative phenomenological analysis [15] to systematically capture the organizational dynamics. The raters will practice with each other in this session. After the first training, the raters will receive audio tapes of prerecorded interviews, which they will independently rate and bring to the second session. During the second session, the ratings will be evaluated, and further questions and uncertainties will be discussed. Furthermore, the interviewers will conduct a training interview, which will be audio-recorded and rated by all involved researchers of the team for feedback.

### Recruitment

Three companies will be recruited through direct contact and researcher networks. At least 10 employees per company, representing different hierarchical levels and workspaces, will be asked to participate. The recruitment process begins with the chief executive officers or human resources management, followed by direct outreach to employees through personal contact, email, and public notice on boards. Management will facilitate initial contact, but participant information will remain confidential. Recruitment materials include the project’s homepage, a white paper, and an information sheet. Participants will receive detailed information about the study, including potential risks and their right to withdraw at any time without consequences. Additionally, participants are informed about data security and their participant rights. Study personnel will then obtain written informed consent.

### Data Collection

The interview will be conducted (and analyzed) in German with each of the three interviewers taking turns. The other interviewers will not be present during the interview but will rate it independently afterward. The interviews will be audio-recorded only. This decision was made in consideration of data protection requests of participating companies and concerns regarding identifiability of employees. Nonverbal and interactional cues will nevertheless be systematically captured by the interviewer through structured observer notes in the research logbook. In order to assess the influence of the lack of some nonverbal cues (gestures, facial expressions, body language, etc) on the rating of the interviews, the coding of the interviewer will be compared for significant discrepancies with the coding of the two independent nonpresent raters.

Data collection will continue until at least 10 interviews have been conducted, and afterward until information saturation is reached, defined as the point where no new themes emerge and the evaluation process between interviews becomes predictable. Theme saturation is considered achieved when the same themes are repeated in at least 2 consecutive interviews without the emergence of any new relevant themes.

### Materials

#### Interview Guide

The interview is based on a semistructured interview guide previously constructed and tested for feasibility. The interview guide has been designed to facilitate a flowing, natural conversation, which lasts about 1 hour. The guide is structured into 5 sections. The first section focuses on the current challenges in the organization, their development and impact as well as resources and barriers for overcoming the challenges. The second section focuses on relational patterns and dynamics in the organization, particularly on how organizational challenges are influenced by these patterns. The third section focuses on the historical development of the organization including insights into the impact of important figures and events in its history. The final section focuses on the level of inner functioning. This encompasses, among other things, the organization’s ability to reflect on itself, deal with critical situations, feedback culture, communicational capabilities, the ability to form healthy and steady working relationships, and their flexible use of adaptive and functional defense mechanisms. The interview guide not only includes the specific questions, but also provides contextual information and what to observe during the interview. The interviewees are encouraged to describe their perspectives and experiences in the organization through examples of interactions and situations.

#### Scoring Sheets

The coding sheets contain 124 coding units that need to be coded for each interview. The coding units are sectioned in respect to the main dimensions of the SyMOA construct. Most coding involves ratings on 3- or 4-point scales. If the raters feel that they do not have sufficient information for the rating of the coding unit, they have the option to leave the coding blank, and it will be treated as missing data in the analysis.

#### Coding Book

All coding units are detailed in a coding book with detailed information on dimension category, definition of coding unit, inclusion and exclusion criteria, detailed information on rating criteria, and anchor examples. The coding book will be prepared in advance. The coding of the first interviews will be used to go through all ratings of the three raters, discuss discrepancies in coding, and clarify and refine the coding criteria. After this initial process, the coding book will remain fixed and is the basis for the coding of all subsequent interviews in the study.

### Data Analysis and Measures

Each dimension has a scoring sheet for evaluation and documentation (see the Materials section). For the quantifiable constructs, the scoring sheets list the operationalized items connected to the questions and have options for ratings on a scale from 0 to 3. Furthermore, the scoring sheets have free text spaces for more descriptive evaluations. Each of the three raters will code and analyze the interview independently, and results will not be discussed with each other until after the data collection is completed. Each interview is analyzed consecutively. After completion of data collection, the scoring sheets for each interview will be analyzed for intercoder reliability and the final analysis data will be used to evaluate SyMOA’s validity. Furthermore, the interviewers as well as the interviewees will be asked to give feedback on the feasibility and acceptability of the interview through a short feedback survey.

*Intercoder reliability* will be tested by coding agreement of the three raters using Krippendorff α in MAXQDA24. This statistical measure will assess coding consistency across evaluators, ensuring the robustness of the SyMOA framework in capturing organizational dynamics. In order to assess the influence of the presence during the interview and being part of the interview interactional dynamic, the coding of the interviewer will be compared with the coding of the two independent raters. This comparison is an exploratory concordance analysis and is not a part of the primary interrater reliability estimate. The analysis levels will be the following:

- Primary intercoder reliability:
  - Alpha (Rater1, Rater2) → Intercoder Reliability of two independent coders as the primary index (*α*≥.80, good; .67-.79, acceptable)
- Secondary bias analysis and consistency exploration:
  - Alpha (R1, Interviewer) → Bias-Analysis 1
  - Alpha (R2, Interviewer) → Bias-Analysis 2
  - Alpha (R1, R2, Interviewer) → Consistency (explorative)

To ensure methodological rigor, the study evaluates various forms of validity [16].

So far, there is no existing gold standard for an operationalized organizational psychodynamic instrument in Germany; therefore, *construct validity* will be evaluated through theory-based and expert-informed approaches rather than through criterion validity. It is examined by evaluating whether the dimensions operationalized in SyMOA are representative and encompassing of systems-psychodynamic organizational theory and diagnostics. This is done through theoretical coherence validation, construct representation, and known-groups validity. *Theoretical validity* is established by assessing whether SyMOA’s findings align with or contribute to existing theoretical frameworks in organizational diagnostics. This will be done by a critical discussion of SyMOA’s results on the background of the current literature on systems-psychodynamics and how it is in line with this literature or may even further develop and contribute to the literature. Furthermore, there will be a critical discussion of SyMOA in relation to other established organizational diagnostics, how it contributes to organizational diagnostic theory, and the merits and the limits of SyMOA in comparison to other organizational diagnostics. The first step is a top-down theoretical mapping*,* comparing the operationalized SyMOA dimensions with core systems-psychodynamic concepts (eg, defense mechanisms). A mapping matrix will be used to document conceptual matches, partial matches, and conceptual gaps.

The second step is a bottom-up *construct representation* analysis following a combined framework from Messick [17,18] unified validity theory and qualitative validity approach by Whittemore et al [19] and Maxwell [20]. Emergent patterns identified in the coded interviews will be grouped into themes and compared with established theoretical constructs of SyMOA to determine whether the material aligns with or diverges from the framework.

The third step of theoretical validity assessment will be a comparative analysis of frameworks [21,22]. Instruments of well-established classical organizational assessment, systems-psychodynamic organizational assessment, and qualitative interview methods will be chosen for comparison. A matrix including key concepts and aspects representing systems-psychodynamics (eg, interactional patterns, conflicts, defense mechanisms, etc), as well as standardization, objectivity, and quantification, will be used to rate each instrument on whether it captures, partially captures, or does not capture these aspects.

Furthermore, *known-groups validity* as an additional form of construct validity will be applied. Based on theoretical assumptions, a valid instrument should be able to differentiate between groups that are expected to exhibit distinct psychodynamic profiles (eg, inner level of functioning or teams with differing conflict levels). Therefore, the SyMOA score profiles for the 3 organizations will be compared according to their ability to capture the theoretically predictable differences across these groups. Evidence of differences consistent with theoretical assumptions may be interpreted as discriminant support for construct validity.

*Expert validity* as a source of evidence for *content validity* will be assessed through feedback from at least five specialists in systems-psychodynamic organizational research, who will evaluate whether SyMOA adequately captures relevant organizational dynamics. The limited number of experts reflects the scarcity of specialists in this field in Germany. Experts will be given the conceptual background of SyMOA, the interview guide as well as the rating sheets. They will be asked to fill in a feedback form, asking among other things about the appropriateness of the interview guide questions, the understandability, the completeness with which the questions represent the construct, the order of the questions, and if any important questions are missing. The feedback from the experts will be used to revise the interview guide.

*External validity* is tested by assessing how well SyMOA’s diagnostic results correspond to measurable organizational factors such as employee turnover, sick days, and conflict rates (reported complaints or conflict cases to human resources, at the leadership level, or to an ombudsman). For analysis of correspondence to these data, the interview data will be grouped into thematic categories given by SyMOA’s construct structure (eg, “organizational regulational capscities,” “communicative capacities,” “organizational relationship pattern,” etc). Frequencies or intensity values will be assigned according to how often a topic was mentioned by each interviewee and how strongly it was rated, thus creating ordinal scaled data. Sick days, conflict rates, and fluctuation will be asserted on the same level of analysis (department and team). A Spearman correlation will be calculated with the 2 data sets. A moderate correlation and argument for external validity would be a Spearman ρ between 0.3 and 0.5.

*Process validation* is ensured through detailed documentation of data collection, analysis, and interpretation procedures to ensure reliability and transparency of the procedure. The documentation system consists of a logbook with a template for the coding process, a template for reflection processes, and a template for observational data.

*Communicative validation*, or member checking, is incorporated by inviting interview participants to review and confirm interpretations of their interview data to ensure that the conclusions drawn are valid. This is done after the SyMOA analysis is finalized.

*Data triangulation* is applied by combining multiple data sources, including interviews and structured on-site observations, to strengthen construct validity. Observational data will include direct observations of employees in their natural work environments and detailed field notes documenting organizational behaviors and interactions. The documentation of the observational data is also based on the 3 dimensions and constructs of SyMOA. However, direct observation focuses more on the nonverbal, unconscious, interpersonal, and situational aspects that can be less well-expressed during interviews. These observations will serve as supplementary contextual information for the analysis and are documented in the logbook.

### Assessment of Feasibility and Acceptability

Feasibility will be assessed by keeping a recruiting and implementation log including a recruitment feasibility protocol (number of contacted organizations and participants who accepted or declined, with reasons if possible), an interview feasibility protocol (no-show rate to interviews, average interview duration, disturbances, comprehension problems, and completeness of interview implementation) and a coding feasibility protocol (data completeness, technical feasibility [incidents that prevented implementation of interview or rating by all raters], reachability of saturation, problems with coding instructions, and ambiguity in coding).

Acceptability will be assessed on the level of the organization, the participants, and the interviewers. The interview participants will receive a short survey asking about the comprehensibility of the questions, how they experienced the atmosphere during the interview, the overall load of the interview, and the appropriateness of the questions for understanding the organization and its challenges. Acceptability on the level of the organization will be assessed by member check and feedback of results to the organization. Finally, the acceptability on the level of the interviewers will be assessed by a rating of the interviewers after each interview and a rating of the feasibility of conducting the interview in the given timeframe, whether the questions were sufficient to allow a deeper understanding of the interviewee’s experience, if normal conversational flow was possible, and if they could ask all questions.

### Data Management and Data Protection

In this study, personal data will be collected in the form of audio recordings of interviews, signed informed consent forms, and interview transcripts. These do not include health-related information, nor will any sensitive internal company data be requested. The audio recordings will be stored password-protected, with names and company identifiers removed. However, complete anonymization is not possible, as voices and interview content may still allow identification. The transcriptions will be pseudonymized so that they can only be attributed to the participants via an externally created ID list. All data will be stored on a university hospital server that complies with General Data Protection Regulation. Participation is voluntary, and consent for data processing can be withdrawn at any time without providing a reason or facing any consequences. Upon withdrawal, no further data will be collected.

### Dissemination Plans

The results of the study will be made public using channels such as peer-reviewed publication and presentation in national and international conferences. The participating organizations will receive oral and written feedback on the results of the interviews with particular care that the feedback is not traceable to the individual interview partner, that is, employees of the organization. The study results will be published in a scientific journal in a timely manner.

## Results

Recruitment and interviewer or coder training began in May 2025 and are expected to be completed by July 2025. Data collection is scheduled to take place between September 2025 to February 2026, followed by data analysis and reporting from February 2026 to April 2026. As this is a study protocol, no study results are available at this stage.

## Discussion

### Principal Findings

This study protocol describes a qualitative validation study of the SyMOA, a systems-psychodynamic diagnostic tool designed to identify organizational challenges, inner functioning, relational dynamics, and unconscious conflict patterns. The planned study is expected to enhance our knowledge of the feasibility of administering SyMOA in organizational settings, the validity of the semistructured interview, and the consistency with which different coders can apply the coding framework. Although no results are available yet, the study is designed to examine whether the operationalized dimensions of SyMOA are meaningful and can be reliably identified by different raters in different organizational contexts.

### Comparison to Prior Work

Most existing assessments of the organizational context focus on directly observable concepts, such as structures, climate, or leadership, but rarely integrate deeper levels of analysis derived from an understanding of unconscious dynamics, relational patterns, and inner-systemic functioning. Systems-psychodynamic research emphasizes the relevance of these aspects, yet there is still a lack of validated diagnostic instruments. SyMOA contributes to this gap by picking up concepts from a clinical instrument (Operationalized Psychodynamic Diagnostics III) and further developing them into an operationalized system that enables a psychodynamic analysis of organizational systems using a semistructured interview guide and a structured coding and rating protocol grounded in systems-psychodynamic theory. This study aims to advance psychodynamic organizational assessment by systematically examining how well such themes and content can be coaxed through a semistructured interview and reliably coded.

### Strengths and Limitations

Strengths of the study include the application of an innovative diagnostic approach based on established psychological diagnostic methods, ensuring both reliability and practical relevance. A further strength of this study protocol is the implementation of multiple validity assessments—including content, construct and external validity, process validation, communicative validation, and researcher triangulation. The validity assessments are complemented with intercoder reliability testing. Observer notes and on-site observations enhance methodological rigor. The use of structured rater training, rating protocols, and a detailed coding book further enhances transparency and reproducibility.

However, certain limitations must be acknowledged. While the study aims to provide evidence, the reliance on qualitative interview data introduces the possibility of subjective interpretation, which data and researcher triangulation are designed to limit. Another limitation is the extent to which interpretative qualitative data can be contextualized with external quantitative data to assess the external validity of the construct. Furthermore, the determination of external validity will be limited as the assessment relies on correlations with staff turnover, sick days, and conflict data. However, these indicators may be influenced by various external factors (eg, economic conditions and industry trends) that are not accounted for in the analysis. In addition, a potential selection bias in the recruitment process for the interviews could occur if certain characteristics of the employees (eg, attitudes, greater self-reflection, or time constraints) influence their willingness to participate. With regard to the interview data, a potential resistance or social desirability bias can be expected due to fear of repercussions or a desire to present the organization in a positive light. The use of audio recordings only as the basis for the coding process limits access to nonverbal cues that may be important for accurate interpretation of the interviews. These limitations will be addressed through triangulation, structured observer documentation, and consistency protocols, and a bias analysis between the interviewer coding and the two independent raters. This analysis is not included in the primary intercoder reliability analysis, but the comparison can reveal systematic differences. Such differences possibly reflect richer contextual understanding when present in the situation. Nevertheless, the limitations remain important considerations for interpreting the findings. Finally, the study will collect data from 3 organizations which may restrict generalizability.

### Future Directions

The results of this study will guide refinement of the SyMOA interview guide and the coding framework. Future research may include application in diverse sectors, quantitative validation components, and integration with organizational outcome measures.

## Abbreviations

SME: small- and medium-sized enterprise
SyMOA: Systematic Multidimensional Organizational Assessment
